# Tumor Escape Phenotype in Bladder Cancer Is Associated with Loss of HLA Class I Expression, T-Cell Exclusion and Stromal Changes

**DOI:** 10.3390/ijms22147248

**Published:** 2021-07-06

**Authors:** Hernani Gil-Julio, Francisco Perea, Antonio Rodriguez-Nicolas, Jose Manuel Cozar, Amanda Rocío González-Ramirez, Angel Concha, Federico Garrido, Natalia Aptsiauri, Francisco Ruiz-Cabello

**Affiliations:** 1 Servicio de Urología, Hospital Universitario Virgen de las Nieves, 18014 Granada, Spain; hernanij@yahoo.com (H.G.-J.); cozarjm@yahoo.es (J.M.C.); 2 Servicio de Análisis Clínicos, Hospital Universitario Virgen de las Nieves, 18014 Granada, Spain; franciscoj.perea.garcia@juntadeandalucia.es (F.P.); antoniorn87@gmail.com (A.R.-N.); federico.garrido.sspa@juntadeandalucia.es (F.G.); 3 Instituto de Investigación Biosanitaria (ibs.GRANADA), 18014 Granada, Spain; agonzalez@fibao.es; 4 Instituto de Investigación en Urología (IDI-URO), 28035 Madrid, Spain; 5 Hospital Universitario San Cecilio, 18014 Granada, Spain; 6 Departamento de Bioquímica y Biología Molecular e Inmunología III, Universidad de Granada, 18014 Granada, Spain; 7 Departamento de Patología, Biobanco de A Coruña, Complejo Hospitalario Universitario A Coruña, 15006 A Coruña, Spain; angel.concha.lopez@sergas.esl; 8 Fundación de Investigación Biosanitaria, ibs Granada, FIBAO, 18014 Granada, Spain

**Keywords:** bladder cancer, HLA class I, cancer immune escape, TILs, T-cell exclusion, PD-L1, cancer associated fibroblasts

## Abstract

Cancer eradication and clinical outcome of immunotherapy depend on tumor cell immunogenicity, including HLA class I (HLA-I) and PD-L1 expression on malignant cells, and on the characteristics of the tumor microenvironment, such as tumor immune infiltration and stromal reaction. Loss of tumor HLA-I is a common mechanism of immune escape from cytotoxic T lymphocytes and is linked to cancer progression and resistance to immunotherapy with the inhibitors of PD-L1/PD-1 signaling. Here we observed that HLA-I loss in bladder tumors is associated with T cell exclusion and tumor encapsulation with stromal elements rich in FAP-positive cells. In addition, PD-L1 upregulation in HLA-I negative tumors demonstrated a correlation with high tumor grade and worse overall- and cancer-specific survival of the patients. These changes define common immuno-morphological signatures compatible with cancer immune escape and acquired resistance to therapeutic interventions across different types of malignancy. They also may contribute to the search of new targets for cancer treatment, such as FAP-expressing cancer-associated fibroblasts, in refractory bladder tumors.

## 1. Introduction

The success of immune checkpoint inhibitors (ICI) depends, at least partially, on the expression of the maximum number of different HLA alleles, conferring a greater ability to present diverse tumor antigens to T cells [1,2,3]. This antigen presentation and T-cell mediated tumor rejection is a common mechanism of anti-cancer immunity. In addition, it has been widely accepted that the degree of tumor infiltration with CD8+ T cells (Tumor-infiltrating lymphocytes, TILs) is also a favorable prognostic factor for tumor rejection and clinical efficacy of immunotherapy. Tumor cells across different types of cancer frequently lose normal HLA-I expression and the ability of stimulate cytotoxic T-cell responses, which make them capable of escaping the immune attack [4,5]. Among the different known molecular mechanisms of HLA-I loss are mutations/chromosomal aberrations in beta2-microglobulin gene *(B2M*), in *HLA* heavy chain genes and in the genes regulating *IFNγ* signaling pathways. These alterations have been observed in a wide range of cancers and are likely to arise under the selective pressure imposed by the immune system [6,7,8,9,10,11]. Previous studies in patients treated with ICI, such as PD-L1/PD-1 or CTLA-4, reported that aberrations in these genes involved in neoantigen presentation play a key role in tumor immune evasion and cancer recurrence [12,13,14]. PD-L1 is an immune checkpoint molecule, a ligand to PD-1 expressed on T-cells. This co-inhibitory receptor can suppress T cell-mediated immune response.

During natural cancer progression and after administration of immunotherapy tumor microenvironment (TME) experiences certain changes, including stromal re-organization [12,15]. It becomes “cold”, with immune cells and cancer associated fibroblasts retained at the margin of a growing tumor. In lung cancer we previously reported a positive association between tumor HLA-I expression and an “inflamed” or “hot” pattern characterized by CD8+ T-cell infiltration within the tumor parenchyma. At the same time, in HLA-I negative tumors CD8+ T cells were largely restricted to the invasive tumor margin and peritumoral stroma (pattern of T-cell exclusion). We call these two immuno-morphological phenotypes as “permissive” (“hot” or “inflamed” phenotype characterized by the presence of TILs) and “non-permissive” (“cold” or “non-inflamed” phenotype, characterized by T-cell exclusion and tumor encapsulation with the stroma). A simultaneous analysis of tumor HLA-I and PD-L1 expression, together with the evaluation of the density and patterns of TILs, provide an important predictive marker for lung cancer progression and response to ICI [2,3,14,15,16,17]. We previously obtained similar results in bladder cancer [18] and here we further analyze the interaction of tumor cells with the stromal elements, including cancer associated fibroblasts, in the context of tumor immune infiltration and tumor HLA-I and PD-L1 expression.

Frequently, tumor progression creates an immunosuppressive microenvironment with reactive stroma positive for fibroblast activation protein (FAP) and dense extracellular matrix that forms a barrier to the immune cells (NK and CD8+ T-cells) preventing direct contact with tumor cells [15,19]. Previous studies have reported the prognostic value of FAP expression in different tumors and its vital role in tumor invasion and metastasis. This “cold” tumor immunophenotype with T-cell exclusion has been linked to the primary and acquired resistance to ICI [20,21]. Currently, new approaches are being developed to target this “T-cell excluded” phenotype and facilitate the infiltration of T cells into the tumor to stimulate effective anti-tumor immunity and tumor regression [22]. This pattern of T-cell exclusion and lack of tumor-infiltrating cytotoxic T-lymphocytes (TILs) could explain the great difference in the success of CAR-T therapy in leukemia and solid tumors [7,17] where CAR-T cells have a direct access to the circulating malignant cells.

The aim of this study was to evaluate the infiltration patterns and the presence of cancer associated fibroblast (FAP+) in bladder tumors in correlation with HLA-I and PD-L1 expression in order to characterize stromal reaction in tumor microenvironment and compare “permissive” and “non-permissive” immunophenotypes in bladder cancer progression in correlation with clinicopathologic variables. This analysis may help to define patterns of cancer immune escape and primary immune resistance.

## 2. Results

### 2.1. HLA and PD-L1 Expression on Tumor Samples in Correlation with Clinicopathologic Variables

**Figure 1:**
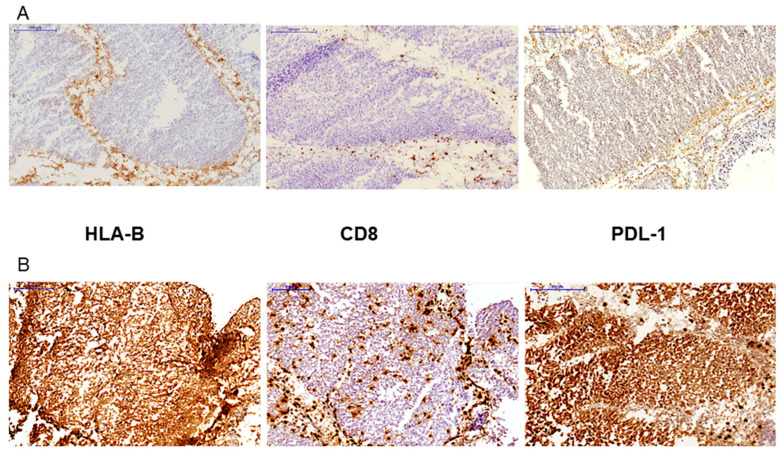
Two examples of tumor immunohistochemistry with different patterns of the expression of HLA class I and PDL-1, and CD8 T-cell infiltration. (**A**) tumor with a HLA-B loss and peritumoral stromal T-cell location (T-cell exclusion pattern); tumor is negative for PD-L1, although there are few positive cells at the tumor margin. (**B**) HLA-I positive tumor (only HLA-B staining is shown) showing intratumoral infiltration with CD8+ T- cells and a homogeneously positive expression of tumor PD-L1. All images are at 100× magnification.

Tumor cell HLA-I expression was evaluated in 131 bladder cancer tissue samples using immunohistological staining with monoclonal antibodies against HLA-ABC, distinct locus-specific monomorphic determinants and against B2M. We detected a significant proportion of tumors with HLA-I altered expression (58 out of 131 samples, (44%) (Table 1) as determined by HLA heavy and light chain immunolabeling with anti-HLA-ABC-B2M complex (W6/32) and anti-B2M (GRH1) antibodies. Out of the 58 tumors with altered HLA-I expression, 23 tumors (39%) demonstrated total HLA-I loss and the remaining 35 tumors (61%) showed a selective loss of HLA-A and/or HLA-B locus (Figure 1). Table 1 summarizes different clinicopathologic parameters and different immuno-morphological tumor characteristics, such as HLA-I and PD-L1 expression, T-cell infiltration patterns and tumor encapsulation and distribution of FAP positive stroma cells. Alterations in tumor HLA expression did not show any correlation with the analyzed clinical variables, such as tumor stage, even when we compared T1/Ta (NMIBC) and T2/3(MIBC) tumors (Table 1).

PD-L1 expression was analyzed in 77 tumor samples. We observed a positive/heterogeneous immunolabeling in 52% of the samples (40 +/het out of 77) (Figure 1). We did not see any correlation between PD-L1 expression and clinicopathologic parameters, including the response to treatment, even when we classified tumors into NMIBC and MIBC subgroups. However, we observed a statistically significant correlation between HLA-I and PD-L1 expression. The majority of HLA-I positive tumors were also positive for PD-L1 expression (*p* = 0.027, Table 2).

**Figure 2:**
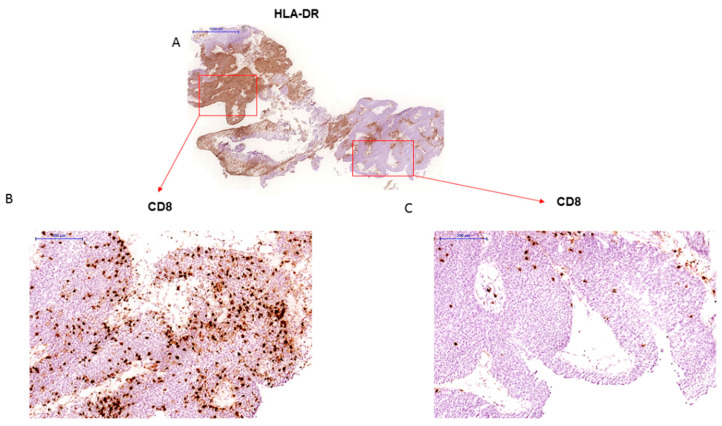
(**A**) Bladder tumor sample with heterogeneous pattern of HLA-DR immunostaining (20× magnification) (**B**) HLA-DR positive area is heavily infiltrated with CD8+ T-cells (“inflamed” tumor), while HLA-DR negative distal zone is significantly less infiltrated with more lymphocytes in the stroma at the tumor margin (**C**) (A and C–at a 100× magnification).

We also evaluated HLA class II expression in 131 bladder tumors samples using anti-HLA-DR monoclonal antibodies. We found positive HLA-II expression in 17 out of 131 tumors (13%); the immunolabeling pattern was mostly heterogeneous, with both positive and negative areas within the same tumor sample. We also observed a correlation between tumor HLA-II expression and the immune infiltration pattern. Most of HLA-II positive tumor samples showed a high degree of infiltration with TILs (*p* = 0.048, Figure 2, Table 3 and Table 4). Many of these tumors were also positive for HLA-I, although this correlation did not reach a statistical significance. There was no association between HLA-II expression and clinicopathologic variables (not shown).

### 2.2. HLA Class I and PD-L1 Expression Is Associated with Distinct T Cell Infiltration Patterns and Stromal Changes with Distinct Distribution of FAP-Expressing Fibroblasts

**Figure 3:**
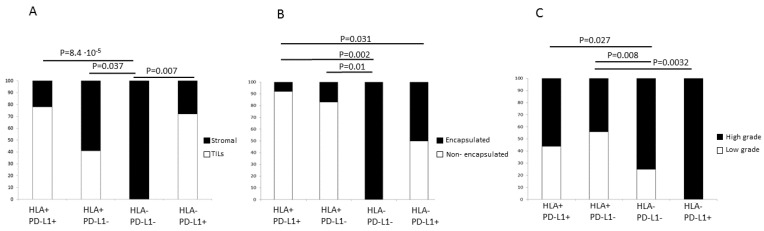
Correlation between HLA-I/PD-L1 tumor phenotype and lymphocyte infiltration (**A**), tumor encapsulation (**B**), and tumor grade (**C**). T-cell exclusion (encapsulated) pattern is typical for HLA-I negative tumors with potentially immunoresistant phenotypes HLA-I-/PD-L1- and HLA-I/-PD-L1+ and an increased presence of FAP-positive stroma cells. Tumors with a diffuse stromal pattern and low FAP expression are mostly positive for HLA-I. C) The majority of high grade tumors are HLA-I negative independently of the PD-L1 status. *p*-values were obtained using chi-square test with IBM SPSS Statistics Ver.21.0 software. Differences between different groups were considered statistically significant at *p* < 0.05.

We analyzed the composition and localization of tumor leukocyte infiltration, especially of T cells, in all bladder tumors using mAbs against molecules expressed by leukocytes (CD45), T cells (CD3 and CD8). We did not find any association between the infiltration pattern and the clinico-morphological parameters. However, when we compared immune infiltration between NMIBC and MIBC tumors, we found that MIBC tumors showed greater degree of infiltration with CD+8 T cells (TILs pattern) (*p* = 0.002) (Table 1). Overall, the degree of the infiltration with CD3+ T-cells and CD8+ T-cells varied among analyzed tumors. Some tumor samples were practically without any infiltration (“cold” tumors with “T-cell excluded” phenotype) and other tumors were heavily infiltrated with T cells (“hot” tumors with “immune-inflamed” phenotype). Expression of HLA-I antigens was strongly correlated with the pattern and grade of T-cell infiltration. Most of tumors with positive HLA-I expression (85%) showed significant intratumoral CD8+ T-cell infiltration, while 69% of tumors with HLA-I alterations (both total and selective HLA loss) had a peritumoral localization of CD8+ T cells (*p* = 6.44 × 10^−10^, Figure 1 and Figure 3, Table 4).

Similarly, tumors with positive/heterogeneous PD-L1 expression showed significant infiltration with CD8+ T-cells and the majority of the PD-L1 negative tumors demonstrated a stromal infiltration pattern (*p* = 1.8 × 10^−5^, Table 4).

With respect to the expression of HLA class II, there was a tendency towards a positive correlation with TILs (Figure 2). However, because of the low number of cases included in this study, the statistical significance was not very high (*p* = 0.048, Table 4). Remarkably, when we analyzed HLA-I and PD-L1 simultaneously, we observed that in double positive tumors (HLA-I+/PD-L1+) the incidence of intratumoral T-cell infiltration is significantly higher than in double negative tumors (HLA-I-/PD-L1-) (*p* = 8.4 × 10^−5^, Figure 3A). Indeed, most of the tumors with negative HLA-I expression and tendency to evade the T-cell immunity had high grade (Figure 3C). In fact, none of the double negative tumors (HLA-I-/PD-L1-) had TILs (Figure 3A) and all of them were high grade (Figure 3C). We also noted that upregulation of PD-L1 in HLA-I negative tumors is associated with a higher degree of infiltration, but these T-cells most likely are inactivated by the inhibitory PD-L1/PD-1 signaling and it could represent an additional route of tumor evasion even in the presence of TILs.

**Figure 4:**
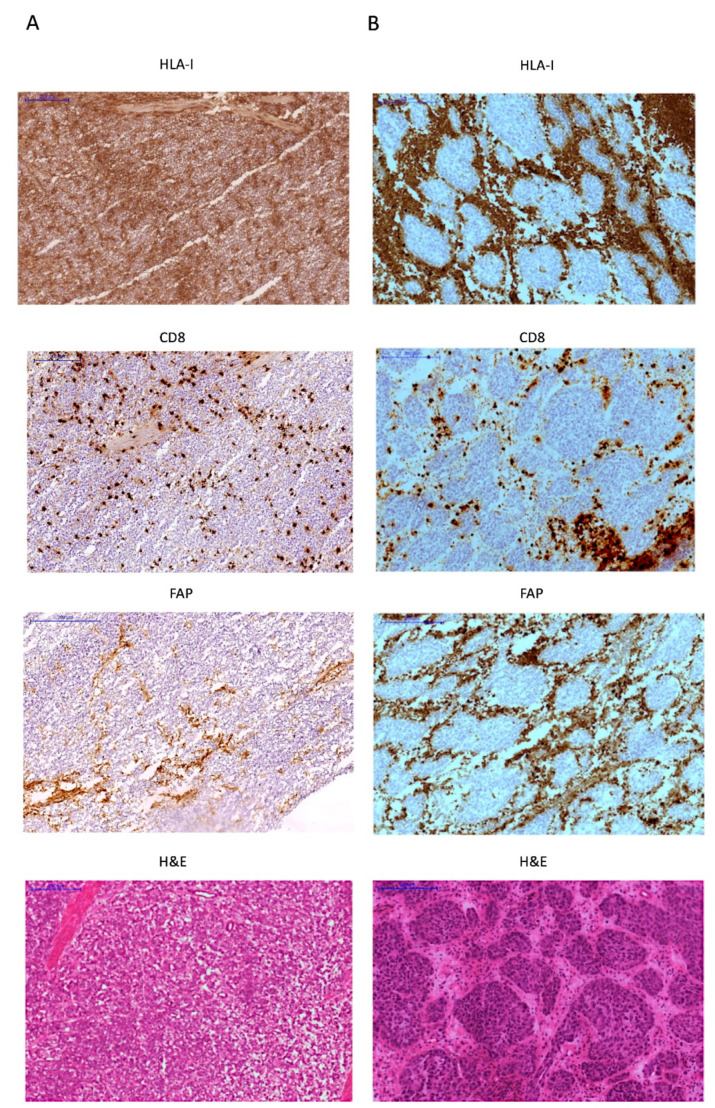
Two different examples of FAP expression in the stroma in high grade tumors. (**A**) HLA-I/PD-L1 double positive tumor heavily infiltrated with T-cells, with few FAP+ stroma cells diffusely distributed in the tumor (**B**) HLA-I negative/PD-L1 positive “encapsulated” tumor with abundant FAP+ cells surrounding HLA-I negative tumor nest and CD8+ T-cells confined in the peritumoral stroma. All images are at 100× magnification.

We also evaluated stromal changes and expression of FAP in 54 tumors. Based on the frequency and tissue distribution of FAP+ fibroblasts, we classified bladder tumors into two groups: non-encapsulated tumors with diffuse distribution of scarce FAP+ fibroblasts and encapsulated tumors with the stroma rich in FAP+ fibroblasts (Figure 4). We did not find any clear correlation between tumor encapsulation, the presence of FAP-expressing stromal fibroblasts and clinicopathologic parameters (Table 1).

We found that the majority of HLA-positive tumors have diffuse distribution of FAP+ fibroblasts in the stroma within the tumor mass, with only 7% of them demonstrating a peritumoral stromal capsule. In contrast, the incidence of encapsulated tumors was around 36% in tumors with altered HLA-I expression (*p* = 0.008, Table 5). In addition, the encapsulated morphology showed a significantly correlation with the T-cell exclusion pattern (*p* = 0.027, Table 5, Figure 4) and increased density of FAP+ cells at the tumor margin. On the contrary, most of the non-encapsulated tumors were heavily infiltrated with TILs (“inflamed” tumors). When the expression of HLA-I and PD-L1 was analyzed simultaneously, we observed once again that HLA-I expression has a stronger link with the tumor immune phenotype and the stroma reaction. Most of the HLA-I positive tumors have non-encapsulated structure with diffuse and scarce FAP+ stroma cells. The incidence of encapsulated tumors is higher in the tumors negative for HLA-I and positive for PD-L1 (*p* = 0.031 Figure 3B), and reaches the maximum in HLA-I/PD-L1 double negative cases (*p* = 0.002 Figure 3B). Therefore, PD-L1 upregulation in HLA-I negative tumors is associated with a slight increase in lymphocyte infiltration and a decrease in the incidence of the encapsulated structure. It is likely that tumors with these types of immune-morphological patterns use two mechanisms of immune evasion: loss of HLA-I and activation of the T-cell inhibitory signaling by upregulation of PD-L1.

### 2.3. Analysis of HLA-I/PD-L1 Tumor Phenotypes and Patient Survival

The overall and cancer-specific survival analysis in patients with different tumor HLA-I and PD-L1 co-expression patterns (77 tumors analyzed) (HLA-I/PD-L1 double positive (23 tumors); HLA-I positive/PD-L1 negative (12 tumors) HLA-I negative/PD-L1 positive (17 tumors); and HLA-I/PD-L1 double negative (25 tumors), did not reveal any statistically significant differences between survival times in these groups (LogRank (Mantel-Cox) 7257; *p* = 0.064). However, when HLA-I positive/PD-L1 negative and HLA-I negative/PD-L1 positive cases were analyzed independently, we could see that the average survival time of patients with HLA-I positive/PD-L1 negative tumor was longer (161 months) than that in patients with HLA-I negative/PD-L1 positive tumors (only 54 months) (Log Rank (Mantel-Cox) 5.18; *p* = 0.023). Overall survival in this last analysis averaged 152.5 months. We also found that double positive tumors showed a statistically significant higher survival than HLA-I negative/PD-L1 positive tumors (*p* = 0.049). In addition, cancer-specific survival was also higher in patients with HLA-I positive/PD-L1 negative tumors (*p* = 0.039) and even more significant in double positive tumors (*p* = 0.019) when compared to the tumors with immune escape phenotype HLA-I negative/PD-L1 positive (Figure S1). However, when we subdivided the patient cohort into smaller subgroups based on the tumor immunophenotype and performed a multivariate analysis adding other variables to the Cox Regression model, this statistical significance was lost; perhaps due to the low numbers of cases.

### 2.4. LOH at the Chromosome 6 Contribute to the Selective HLA-A/B Locus Loss in Bladder Tumors

**Figure 5:**
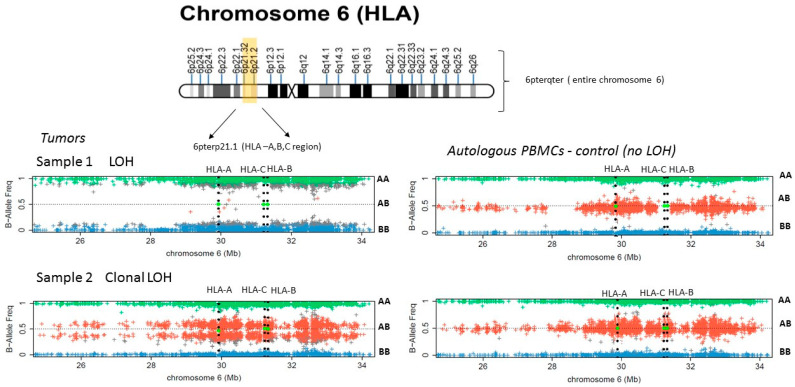
SNPs array analysis of LOH in bladder tumors with alterations in HLA-I expression. Sample 1 picture depicts LOH involving entire *HLA-A*, *B*, *C* genetic region (6p21) (deletion 6pter21.1, as demonstrated on the chromosome 6 map) in the tumor sample, as compared to a normal pattern in autologous PBMCs. Sample 2 plot depicts a clonal LOH of the entire chromosome 6 (6pterqter) in the tumor, and affects only a proportion of tumor cells (two red bands of SNPs). AA, AB, BB-represent the different genotypes corresponding to homozygous (AA or BB) and heterozygous (AB) versions.

We performed an SNPs analysis to detect LOH in the *HLA* region of chromosome 6 and HLA genotyping using DNA from five microdissected bladder tumor samples and DNA extracted from autologous peripheral blood mononuclear cells (PBMCs). These tumors had different types of HLA-I alterations as demonstrated by immunohistological analysis. Three of the cases did not express either HLA-A (samples 1, 2 in Figure 5 and Figure S2) or HLA-B (not shown). In one case we observed a complete loss of HLA-ABC/B2M expression (sample 4, Figure S3), and one tumor did not have any alterations based on immunohistological analysis (sample 5, Figure S3). We did not detect LOH-6 in Sample 4 with total HLA-I loss of expression (Figure S3). However, in cases with a selective loss of the HLA-A or B locus, we observed various alterations in chromosome 6 (Figure 5 and Figure S2). In Sample 1 we found a CN-LOH at 6pterp21.1 area of chromosome 6 (Figure 5 and Figure S2) and in Sample 3 we detected a loss of 6pterp21.2. region (not shown), both of which cause a total loss of all *HLA* loci. The somatic origin of the CN-LOH influencing *HLA* genes was confirmed by the comparison with the results obtained in the control (PBMCs) suggesting that the observed large deletions indicative of CN-LOH in *HLA* region represent an acquired aberration in cancer cells. In Sample 2, the data of the SNP analysis demonstrate a heterogeneous clonal pattern with CN-LOH (6pterqter deletion) that causes the loss of entire chromosome 6 (Figure S2, sample 2). CN-LOH. We estimate that the subclone with CN-LOH (6pterqter) approximately represents 20–25% of the cells. We cannot rule out that this tumor subclone is in fact underrepresented and that, at least in part, it reflects the presence of normal non-malignant cells from the microdissected tumor tissue sample.

## 3. Discussion

Many urothelial cancer patients do not benefit from ICIs treatment and the mechanisms of the resistance remains incompletely understood. It is believed that a combination of factors, including altered HLA expression, tumor PD-L1 upregulation, somatic mutations with variable neoantigen load, the degree and pattern of the immune infiltration (T-cell exclusion or intratumoral infiltration), all contribute to the observed poor clinical efficacy of ICI in many cancers [23,24]. In this study a significant proportion of bladder tumors (44%) demonstrated total and partial HLA-I alterations. In particular, we observed a high incidence of selective HLA-A or HLA-B locus losses. Our data of selective HLA loss expression is surprisingly high compared to that observed in other tumors [25,26,27]. They could be even higher if the presence of somatic *HLA-A*, *B*, *C* allelic mutation had also been studied [28]. Although a downregulation of HLA-I allows tumor cells to escape immune detection by cytotoxic T-cells, complete HLA-I loss makes cells susceptible to NK cell cytotoxicity. In this sense, it has been suggested that the decreased expression or selective HLA-I loss, rather than complete absence, can allow tumors to escape from both T-cell and NK surveillance, because cell surface HLA-I molecules provide an inhibitory signal to natural killer cells [13,29]. Some reports suggest that an epigenetic reversible mechanism, rather than genetic HLA defects, are responsible for HLA-I downregulation in tumors [3]. In this study, we did not detect LOH-6 in a tumor with total HLA loss detected by immunohistochemistry (Figure S3) suggesting that the cause of this loss may be associated with epigenetic and/or transcriptional dysregulation of HLA expression. We have previously reported that a coordinated downregulation of antigen processing machinery genes without any alterations in *B2M* gene is responsible for HLA-I loss in bladder tumors [30]. Here we further demonstrate that some selective HLA locus losses can be linked to the LOH in *HLA* genes. This haplotype loss could explain the loss of one allele. The remaining allele could be suppressed by other alternative mechanism (i.e., epigenetic or transcriptional downregulation). This molecular event may be prevalent in bladder carcinoma. This molecular mechanism has been frequently observed in others types of human cancers and may constitute a universal immune escape strategy with important clinical implication for primary and secondary resistance to ICIs [14,17,25].

In relation to the expression of HLA-II, we noticed that most tumors with positive HLA-II expression are also positive for HLA-I and have a pattern of “inflamed tumor” with the presence of TILs (Figure 2 and Table 3). In addition, we found that the pattern of T cell-exclusion is associated with partial or total loss of HLA-I expression (Figure 1, Figure 2, Figure 3 and Figure 4; Table 4 and Table 5). In these cases, CD8+ T cells were confined in the fibroblast- and collagen-rich peritumoural stroma, unable to reach tumor cells. In addition, our results revealed that the expression of PD-L1 in bladder cancer has a positive correlation with HLA-I expression. It suggests that tumors that do not lose HLA-I during cancer development could use the expression PD-L1 to escape cytotoxic responses.

An important finding of this study is that both tumor HLA-I and PD-L1 expression are associated with the “inflamed” tumor phenotype, intratumoral infiltration with CD8+ T-cell and low tumor grade, indicative of a better prognosis (Figure 3). In addition, we discovered that the majority of the tumors with immune escape phenotype (HLA-I-/PD-L1+) had a high grade. We also argue that the observed different stromal pattern evolution can condition both the expression of HLA and T-cell infiltration. We have observed a close association be-tween the distribution and presence of FAP+ fibroblast and the pattern of immune infiltration (Figure 4). In our study, the highest incidence of FAP+ cells were observed in tumors surrounded by a stroma, which prevents the migration of the immune cells into the tumor (T-cell exclusion pattern). These types of tumor frequently have a significant downregulation of HLA expression. In contrast, tumors with few FAP+ cells exhibit an inflamed phenotype with TILs (Figure 4, Table 5). These phenotypes may in some cases represent different phases of a continuous process of tumor immunoediting [15,19], from HLA-I positive tumor to a HLA-I negative phenotype. In support of this multistep process, Zaretsky and colleagues showed that biopsies obtained at the time of response to the treatment had a marked increase in intratumoral CD8+ T-cell infiltrate density, whereas relapsed lesions showed an immune excluded state with CD8+ T cells largely restricted to the invasive tumor margin [12]. Here we were able to see that within the same tumor sample, different areas with different HLA class I and II expression demonstrate different infiltration pattern: HLA positive areas heavily infiltrated with TILs, and HLA-low areas with an immune excluded pattern and only scarce T cells (Figure 2).

The mechanisms of T-cell exclusion are not completely understood. However, they might be linked to the activation of several molecular mechanisms that impair priming of tumor-specific T cells, suppress T cell infiltration and promote resistance to ICIs. Non-inflamed tumors without TILs have been associated to activated *β-catenin* and *PPAR-g* [20,31], increased production of transforming growth factor β (TGF-β), presence of cancer associated fibroblasts expressing FAP protein [32] and loss of *PTEN* [33]. These cells promote an immunosuppressive microenvironment through the induction and accumulation of pro-tumoral macrophages [17,34], activation of the TGF-β pathway and production of IL-10, all of which induce a profound downregulation of HLA-I [35].

All these factors could contribute to the immune escape of double negative tumors and HLA-I negative/PD-L1 positive tumors. Therefore, we were not surprised to see that these two tumor phenotypes had a higher grade (Figure 3). In particular, an immune escape tumor phenotype (HLA-I-/PD-L1+) was linked to a decreased overall survival (OS) and cancer-specific survival (CSS) (Figure S1). There is evidence indicating that cancer-associated fibroblasts promote the EMT and are also associated with tumor progression and resistance to therapy [32,36]. Interestingly, administration of TGF-β blocking antibody together with anti-PD-L1 antibodies in a mouse model facilitated T-cell infiltration into the center of the tumor and provoked a strong anti-tumor immunity and tumor regression [22]. This treatment protocol can be attractive when tumor HLA-I downregulation is caused by transcriptional dysregulation [3] and there are no structural alterations in *HLA* genes or LOH in HLA genomic regions [17,37]. In this context, we have data suggesting that LOH-6 affecting *HLA* genes is a very common molecular defect in bladder tumors and bladder cancer cell lines (Garrido et al., un-published results), with a significant fraction of bladder cancer cell lines showing CN-LOH 6pterqter pattern corresponding to whole-genome doubling (WGD), involving the duplication of a complete set of chromosomes [38,39]. We believe that, at least in some tumors with encapsulated pattern and total HLA-I loss, regulatory mechanisms of HLA-I downregulation prevail and that it could be reversed by immunomodulatory treatment (sample 4, Figure S3). Alternatively, total HLA-I loss could represent a consequence of a combination of two mechanisms: LOH-6 in HLA region and transcriptional downregulation of HLA expression caused by cytokines, which would obviously be an obstacle for cancer immunotherapy and ICIs strategies (Samples 1 and 2, Figure 5 and Figure S2). In fact, we previously observed an increase in the incidence of chromosomal aberrations at chromosome 6 (LOH-6) that involves HLA region in recurrent bladder tumors after BCG immunotherapy, suggesting an importance of T cell immunoselection during immunotherapy [37]. However, in this study only five cases were analyzed for LOH and none of the studied cases had ICIs therapy. Therefore, we could not evaluate the impact of the LOH in *HLA* genes to the clinical response to the immunotherapy, which represents a limitation of this study. Hence, the obtained results should be confirmed on a larger cohort of patients.

In our study tumor PD-L1 expression alone did not show any association with clinicopathologic characteristics of the studied patients. In fact, published data related to the PD-L1 expression in cancer are someway contradictory. It has been reported that the associations between PD-L1 expression and prognosis vary in different malignancies [40,41]. In urothelial bladder cancer, patients with higher proportion of PD-L1 positive tumor cells had increased recurrence and worse survival following cystectomy [42]. In contrast, a recent study reported that positive tumor PD-L1 expression does not have a predictive value for OS, but positive PD-L1 expression in stromal cells was significantly associated with longer survival in patients who developed metastases and received subsequent chemotherapy [43].

In our study, only a simultaneous analysis of PD-L1 with HLA-I expression yielded results that showed a statistical significance. Importantly, HLA-I negative/PD-L1 positive phenotype, which had a correlation with high tumor grade, showed worse overall and cancer-specific survival than HLA-I positive/PD-L1 negative and double positive tumors, suggesting the importance of these biomarkers on the immune escape and cancer progression. However, when we performed a multivariate analysis adding other parameters in Cox Regression model, this statistical significance was lost. The size of the cohorts for the survival analysis became too small after classification of the patients according to the HLA-I/PD-L1 phenotypes and it represents another limitation of the study. Therefore, our conclusions should be verified in larger series of cases.

## 4. Material and Methods

### 4.1. Patients and Tumor Samples

We studied 131 patients who underwent transurethral resection of bladder (TURB) and analyzed their tumor samples collected from Biobank of Hospital Universitario Virgen de las Nieves (Granada, Spain). Tumor samples were processed and stored at −80 °C until further immunohistological analysis. All patients are Caucasian from Andalusia, a region in southern Spain. Informed consent approved by the Ethics Committee of our institution was signed by the patients included in this study. Previously, all medical records and tumor sections were reviewed by both an urologist and a pathologist. The tumor histological subtype in all cases was urothelial. Tumors were graded according to the World Health Organization classification [44] and staged according American Joint Committee on Cancer (AJCC) Staging Manual, 8th edition [45].

Demographic, clinical and pathological characteristics of the studied patients are summarized in Table 1. Two main group of tumors were included: 88 (68%) patients with non-muscle invasive bladder cancer (NMIBC, Ta + T1) and 43 (32%) with muscle-invasive bladder cancer (MIBC, T2-T3) tumors.

After TURB, all patients who were staged as Ta-T1 were included in a surveillance program due to the risk of recurrence and progression. We have established a post-operative follow up plan that includes a combination of urine cytology, cystoscopy and an ultrasound evaluation conducted at 3-month intervals during the first year, at 6-month intervals during the second year and annually afterwards. Tumor recurrence was considered when a new lesion was detected by cystoscopy and ultrasound evaluation 3 months after the initial tumor resection. Those subjects staged as T2 and higher underwent radical cystectomy or trimodality bladder preservation therapy. The mean follow up time for patient survival was 62.9 months (1–256). Overall survival was defined as the interval between the first TURB and death or last observation. Of the 131 patients, 42 (32%) died, 51 (39%) had tumor recurrence (12 of which died) and 50 (38.2%) survived without recurrence during follow up. Average time of recurrence was 7.55 ± 15.38 months.

77 patients received intravesical therapy based on their initial stage after TURB. Patients diagnosed with a low grade Ta-T1 (LG) bladder cancer (42 patients) were treated with Mitomycin C (MMC). Patients with high grade T1 (HG) bladder cancer (15 patients) received Bacillus Calmette-Guérin (BCG). Moreover, 10 patients received both treatments (Mitomycin C + BCG) starting with Mitomycin and followed by BCG after morphological examination of the tumor.

### 4.2. Immunohistological Analysis of Tumor Samples

We evaluated 131 cryopreserved bladder tumor samples. After transurethral tumor resection tissue samples were immediately stored at −80 °C. Cryosections were obtained using a microtome-cryostat (Bright), allowed to dry at room temperature for 4–18 h, fixed in acetone at 4 °C for 10 min, and stored at −80 °C until immunolabeling with Biotin-Streptavidin kit (supersensitive Multilink HRP/DAB kit, BioGenex, The Hague, The Netherlands). Tissue sections were evaluated by two independent pathologists. We analyzed the following molecules using specific monoclonal antibodies: W6/32-against HLA-A, B, and C heavy chain/B2M complex (a gift from Dr. Bodmer, Imperial Cancer Research Fund Laboratories, London, UK); GRH-1, which recognizes free and heavy-chain-associated B2M (produced and characterized in our laboratory); HC-10-against free HLA-B and C heavy chains (Nordic- MUbio, Rangeerweg, The Netherlands), anti-HLA-A which recognizes a subset of HLA-A locus [46], and 42IB5 against HLA-B locus [47].

PD-L1 expression was analyzed in 77 tumor samples. We used monoclonal anti-PD-L1 antibody (clone 22c3), a corresponding IHC kit for PD-L1 22c3 pharmDx (DAKO), and EnVision FLEX System (DAKO, Santa Clara, CA, USA) on 2.5 μm sections following the manufacturer’s recommendations. PD-L1 immunolabeling was scored using criteria adapted from Eckstein et al. [48] based on partial or complete membranous staining at any intensity with a variably strong component of cytoplasmic staining and was interpreted as: no PD-L1 expression (<5% of positive tumor cells), heterogeneous or low PD-L1 expression (5–25% positive cells) and positive or high PD-L1 expression (>25% of positive cells).

The following cells were excluded from scoring: infiltrating immune cell, normal cells, and non-specific staining of necrotic cells. For statistical analysis tumors with heterogeneous and positive expression were combined together in the same group. We also studied HLA class II expression using GRB1 mAb against HLA-DR molecules (produced and characterized in our laboratory). These mAbs were previously described in [49,50].

Total loss of HLA-I molecules was defined by a negative staining with W6/32 and GRH-1 mAbs according to the criteria established by the HLA and Cancer component of the 1996 International Histocompatibility Workshop [51]. We could distinguish three different expression patterns in the immunolabeled samples: positive (>75% of tumor cells positive), heterogeneous (25–75% positive cells) and negative (<25% positive cells). For statistical analysis heterogeneous and positive tumors were combined together in the same group. We found a proportion of tumors with selective HLA-I loss and for the statistical analysis both total and selective HLA-I loss were classified in the same group with altered HLA-I expression.

To evaluate cryopreserved tumor samples for lymphocyte infiltration we used the following monoclonal antibodies: anti-CD45 (GRT2, produced in our laboratory [49], OKT3 (anti-CD3, ATCC, Teddington, UK), OKT8 (anti-CD8, ATCC). The primary antibody was replaced with PBS in negative controls and no immunolabeling was detected in control preparations. We previously defined two different infiltration patterns based on the localization of immune cells: a stromal pattern defined as a “T-cell exclusion” phenotype with the immune cells retained in the stroma at the invasive tumor margin, and an “immune inflamed” phenotype with TILs, in which T cells are in close contact with malignant cells [17].

We also assessed the expression of fibroblast activation protein (FAP) in bladder tumor microenvironment using anti-FAP antibody (clone F11-24) purchased from Merck KGaA (Darmstadt, Germany). Evaluation of FAP expression was performed based on the positive immunolabeling and a pattern of distribution within the tumor. Tumors with FAP positive fibroblasts and a strong stromal reaction forming a dense capsule around the tumor nest were classified as “encapsulated”. In contrast, tumors with negative FAP expression (absence of cancer-associated fibroblasts) or with few diffusely distributed FAP+ fibroblasts were categorized as “non-encapsulated”. Immunostained tissue sections were further scanned and analyzed using a ‘Panoramic Scanner MIDI II’ and ‘Panoramic Viewer’ (3DHISTECH Ltd., Budapest, Hungary).

### 4.3. Microdissection and DNA Isolation

Cryopreserved tissue sections of 8–10-μm thickness, fixed in 70% ethanol and stained with a 0.05% *w/v* solution of toluidine blue, were microdissected using a laser micromanipulator (PALM MicroLaser Systems, ZEISS, Göttingen, Germany). Microdissected fragments were collected in PALM Adhesive Caps. Tumor DNA was isolated from these fragments using QIAamp Tissue Kit (QIAGEN, Hilden, Germany). In all cases, normal autologous DNA was obtained from peripheral blood lymphocytes (PBLs) using QIAGEN DNA isolation kit following the manufacturer’s protocol.

### 4.4. Loss of Heterozygosity (LOH) in HLA-I Region of Chromosome 6 Analyzed by Single Nucleotide Polymorphism (SNP) Arrays

Evaluation of the LOH in tumor samples was done base on the method described previously [52,53,54]. DNA samples from microdissected bladder tumors and autologous peripheral lymphocytes were genotyped using the Illumina Infinium HTS Assay on the ImmunoArray-24 v2 BeadChip (Illumina^®^) according to manufacturer protocol, which detects over 250,000 SNPs selected based on GWAS of the diseases of the immune system. Data for loss of heterozygosity (LOH) analysis and copy number (CN) analysis were obtained from the Illumina GenomeStudio^®^ Genotyping Module v2.0 Software as “Norm Theta” and “R” values. “Norm Theta” represents the B-allele frequency and “R” the joined fluorescence intensity of both channels. While “Norm Theta” can be interpreted directly to detect LOH, “R” needs to be compared to a standard to detect regions of copy number loss or gain. We used immunochip data from unrelated samples of European ancestry to obtain a median fluorescence value per probe to create such a standard and to subsequently obtain Log R ratios [16,17]. A Log R ratio distribution around zero can be regarded as CN neutral, while chromosomal intervals of mainly positive (or negative) Log R ratios can be interpreted as CN gains (or loss). Chromosomal stretches of B-allele frequencies (BAF) with values of mainly zero or one can be interpreted as LOH. Dots with a value of “1” represents SNPs with the “AA” genotype, those with a value of “0” indicate SNPs with “BB” genotype, and dots at “0.5” represents heterozygous “AB” genotype. When a BAF pattern has all three mentioned above values, the sample is heterozygous (AB) and there is no LOH. Complete loss of all SNPs with the AB genotype indicates regions of LOH. Furthermore, loss of SNPs in the AB together with the absence of the copy number alteration, is indicative of a copy neutral LOH (CN-LOH) and the degree of this deviation corresponds to the presence of clonal cells with CN-LOH. In this case samples can be heterogeneous, representing a mixture of tumor cells with and without LOH. We also used the University of California in Santa Cruz (UCSC, USA) Genome Browser (http://genome.ucsc.edu/, accessed on 31 January 2021) to map and characterize the range of the missing regions in chromosomes 6 and 15 (GRCh38/hg38 Assembly).

### 4.5. Statistical Analysis

All statistical tests were performed with the statistical package for IBM SPSS Statistics Ver.21.0. (IBM Corp., Armonk, NY, USA) Variables with normal distribution were expressed as mean ± standard deviation. Variables with non-normal distribution are expressed as medians and interquartile range. For quantitative variables, when two groups were compared, we used the Student’s *t* test (parametric). Categorical variables, such as sex, tumor all clinical characteristics, HLA and PD-L1 expression, infiltrating pattern and encapsulation were coded in two groups and analyzed using the chi-square (X^2^) or Fisher’s exact test in case when the validity criteria were not reached. Differences were considered statistically significant at *p* < 0.05. 

For the survival analysis, the date of diagnosis was considered as time zero and the date of death as the event. Overall survival estimates were made using the Kaplan-Meier product limit method. To compare survival curves, the log rank test (Log Rank-Mantel Cox) was performed. We used a multivariate Cox Regression model (proportional hazards ratios and 95% CI) to predict the survival of the patients in our study. The value *p* < 0.05 was considered statistically significant.

## 5. Conclusions

In this work we demonstrated a strong association between the tumor loss of HLA-I and the formation of a “non-permissive” tumor microenvironment characterized by reduced immune infiltration and strong stromal reaction with increased presence of FAP+ stroma cells. This could lead to cancer immune escape due to the exclusion of cytotoxic T cell from the tumor mass and upregulation of inhibitory signaling mediated by PD-L1/PD-1 engagement.

We also observed that HLA-I negative/PD-L1 positive phenotype has a significant correlation with high tumor grade and poor overall and cancer-specific survival. In addition, HLA-I and PD-L1 are important determinants of tumor T-cell exclusion and stromal changes associated with tumor development. Similar association we observed in lung cancer [4,16]. All these data suggest that loss of HLA-I and PD-L1 upregulation are essential and common determinants of cancer immune escape in different types of malignancy and might cause primary resistance to ICIs. Thus, a common strategy aimed at modulating tumor microenvironment rich in FAP+ fibroblasts, inhibiting PD-L1 expression, increasing tumor T-cell infiltration and upregulating HLA-I in tumors without LOH or mutations/deletions in *HLA* genes, might provide a new framework for rational combination immunotherapy.

## Figures and Tables

**Table 1:** Clinical Features of bladder cancer patients and tumor HLA class I and PD-L1 expression, infiltration pattern and tumor architecture with FAP+ cell distribution.

Clinical Features	HLA-I Expression			PD-L1 EXPRESSION			Infiltration Pattern			FAP + Distribution		
	Positive *n* = 73 (56%)	Partial/Total Loss *n* = 58 (44%)	*p* *	Positive/Het *n* = 40 (52%)	Negative *n* = 37 (48%)	*p* *	TILs *n* = 78 (60%)	Stromal *n* = 38 (40%)	*p* *	Encapsulated *n* = 11 (20%)	Non-Encapsulated *n* = 43 (80%)	*p* *
Age												
Mean: 69 (22–93)	69.26 ± 9.76	68.55± 15.18	0.759	68.63 ± 14.92	67.68,26 ± 13.58	0.772	70.7 ± 10.15	66.43 ± 15.02	0.081	64.09 ± 19.14	69.53 ± 10.87	0.216
Sex												
Male: 107 (82%)	57 (78%)	50 (86%)	0.232	30 (75%)	32 (87%)	0.204	58 (74%)	47 (92%)	0.011	6 (55%)	34 (79%)	0.129
Female: 24 (18%)	16(22%)	8 (14%)		10 (25%)	5 (13%)		20 (26%)	4 (8%)		5 (45%)	9 (21%)	
Tumor stage												
NMIBC: (Ta + T1): 88 (67%)	46 (63%)	42 (72%)	0.260	31 (78%)	32 (87%)	0.307	44 (56%)	42 (82%)	0.002	8 (73%)	32 (74%)	1.000
MIBC: (T2 + T3): 43 (33%)	27 (37%)	16 (28%)		9 (22%)	5 (13%)		34 (44%)	9 (18%)		3 (27%)	11 (26%)	
Nodal status												
N0: 122 (93%)	68 (93%)	54 (93%)	0.999	36 (90%)	35 (95%)	0.676	70 (90%)	50 (98%)	0.071	9 (82%)	39 (91%)	0.590
N1: 9 (7%)	5 (7%)	4 (7%)		4 (10%)	2 (5%)		8 (10%)	1 (2%)		2 (18%)	4 (9%)	
Metastasis												
M0: 125(95%)	70 (96%)	55 (94%)	0.999	37 (93%)	35 (95%)	1.000	73 (94%)	50 (98%)	0.241	9 (82%)	40 (93%)	0.266
M1: 6 (5%)	3 (4%)	3 (6%)		3 (7%)	2 (5%)		5 (6%)	1 (2%)		2 (18%)	3 (7%)	
Focality												
<3 foci: 84 (64%)	44 (60%)	40 (69%)	0.303	33 (83%)	24 (65%)	0.078	46 (59%)	36 (71%)	0.180	7 (64%)	32 (74%)	0.475
≥3 foci: 47 (36%)	29 (40%)	18 (31%)		7 (17%)	13 (35%)		32 (41%)	15 (29%)		4 (36%)	11 (26%)	
Tumor size												
<3 cm: 54 (41%)	30 (41%)	24 (41%)	0.974	24 (60%)	17 (46%)	0.217	32 (41%)	20 (39%)	0.838	4 (36%)	24 (56%)	0.249
≥3 cm: 77 (59%)	43 (59%)	34 (59%		16 (40%)	20 (54%)		46 (59%)	31 (61%)		7 (64%)	19 (44%)	
Grade												
High grade: 96(73%)	54 (74%)	42 (72%)	0.237	24 (40%)	19 (51%)	0.445	62 (80%)	33 (65%)	0.062	10 (91%)	27 (63%)	0.143
Low grade: 35 (27%)	19 (26%)	16 (28%)		16 (60%)	18 (49%)		16 (20%)	18 (35%)		1 (9%)	16 (37%)	
Events												
Death: 42 (32%)	26 (13%)	16 (36%)	0.328	4 (10%)	3 (8%)	1.000	28 (36%)	14 (28%)	0.317	1 (9%)	4 (9%)	1.000
Recurrence: 51(39%)	32 (44%)	19 (44%)	0.197	14 (35%)	17 (46%)	0.328	30 (39%)	20 (39%)	0.931	3 (27%)	17 (40%)	0.510

* The results were obtained performing chi-square test and when validity conditions were not accomplished we used exact Fisher test with IBM SPSS Statistics Ver.21.0 software. Differences between different groups were considered statistically significant at *p* < 0.05.

**Table 2:** Correlation between HLA-I and PD-L1 expression.

	PD-L1 Expression			
HLA-I Expression	Positive/Het	Negative	Total	*p*-Value *
Positive	23 (66%)	12 (34%)	35	0.027
Total/Partial loss	17 (41%)	25 (59%)	42	

* The results were obtained using chi-square test with IBM SPSS Statistics Ver.21.0 software. Differences between different groups were considered statistically significant at *p* < 0.05.

**Table 3:** Phenotypic characteristics and pattern of T cell infiltration in HLA class II positive tumors.

Sample	HLA-DR	HLA-ABC	B2m	HLA-A	HLA-B	HLA-ABC Free Heavy Chain	PD-L1	Infiltration Pattern (CD8+)
19180164	+	+	+	−	+	+	Het	TILs
VR117	+	+	+	−	−	+	−	Stromal
VR130	+	+	+	−	−	+	−	Stromal
19A028	+	+	+	+	−	−	Het	Stromal
19A029	Het	+	+	+	−	−	Het	TILs
RTU 366	Het	+	+	− *	+	+	−	TILs
RTU 381	+	+	+	+	+	+	Het	TILs
RTU 232	Het	+	+	+	+	+	−	TILs
19170421	Het	+	+	+	+	+	+	Stromal/TILS
19170454	Het	+	+	+	+	+	+	TILs
19180136	Het	+	+	+	+	+	Het	TILs
RTU 739	+	+	+	+	+	+	+	TILs
RTU 040	+	+	+	−	+	+	+	TILs
19050	+	+	+	+	+	+	+	TILs
VE9	+	+	+	+	+	+	ND	TILs
VR58	+	+	+	+	+	+	Het	TILs
VR86	+	+	+	+	+	+	+	TILs

* In this case the antibody does not recognize specific HLA-A alleles of the patient, since even the immunolabeling of the stroma was HLA-A negative; ND: Not done.

**Table 4:** Correlation between HLA-I, HLA-II, PD-L1 expression and T-cell infiltration patterns.

	Infiltration Pattern		Total	*p*-Value *
	TILs	Stromal		
**HLA-I expression**				
Positive	60 (85%)	11 (15%)	71 (56%)	6.44 × 10^−10^
Total/Partial loss	18 (31%)	40 (69%)	58 (44%)	
**HLA-II expression**				
Positive	14 (82%)	3 (18%)	17 (13%)	0.048
Negative	64 (57%)	48 (43%)	112 (87%)	
**PD-LI expression**				
Positive/het	30 (79%)	8 (21%)	38 (51%)	1.8 × 10^−5^
Negative	11 (31%)	26 (70%)	37 (48%)	

* The results were obtained performing chi-square test with IBM SPSS Statistics Ver.21.0 software. Differences between different groups were considered statistically significant at *p* < 0.05.

**Table 5:** Association of tumor encapsulation with HLA-I, PD-L1 expression and T-cell infiltration pattern.

Feature	Encapsulated 11 (20%)	Non-Encapsulated 43 (80%)	*p*-Value *
**HLA-I expression**			
Positive	2 (7%)	27 (93%)	0.008 ^a^
Total/Partial loss	9 (36%)	16 (64%)	
**PD-L1 expression**			
Positive/het	4 (13%)	28 (87%)	0.083 ^b^
Negative	7 (32%)	15 (68%)	
**Infiltration Pattern**			
TILs	4 (11%)	31(89%)	0.027 ^b^
Stromal	7 (41%)	10 (59%)	

* The results were obtained with IBM SPSS Statistics Ver.21.0 software. Differences between different groups were considered statistically significant at *p* < 0.05. ^a^ result was obtained with chi-square test, ^b^ results obtained using Fisher exact test.

## Supplementary Materials

The following are available online at https://www.mdpi.com/article/10.3390/ijms22147248/s1.

## References

[1] Trujillo J.A., Sweis R.F., Bao R., Luke J.J. (2018). T cell–inflamed versus Non-T cell–inflamed tumors: A conceptual framework for cancer immunotherapy drug development and combination therapy selection. Cancer Immunol. Res..

[2] Sade-Feldman M., Jiao Y.J., Chen J.H., Rooney M.S., Barzily-Rokni M., Eliane J.-P., Bjorgaard S.L., Hammond M.R., Vitzthum H., Blackmon S.M. (2017). Resistance to checkpoint blockade therapy through inactivation of antigen presentation. Nat. Commun..

[3] Paulson K.G., Voillet V., McAfee M.S., Hunter D.S., Wagener F.D., Perdicchio M., Valente W.J., Koelle S.J., Church C.D., Vandeven N. (2018). Acquired cancer resistance to combination immunotherapy from transcriptional loss of class I HLA. Nat. Commun..

[4] Aptsiauri N., Ruiz-Cabello F., Garrido F. (2018). The transition from HLA-I positive to HLA-I negative primary tumors: The road to escape from T-cell responses. Curr. Opin. Immunol..

[5] Garrido F., Aptsiauri N., Doorduijn E.M., Garcia Lora A.M., van Hall T. (2016). The urgent need to recover MHC class I in cancers for effective immunotherapy. Curr. Opin. Immunol..

[6] Bernal M., Ruiz-Cabello F., Concha A., Paschen A., Garrido F. (2012). Implication of the β2-microglobulin gene in the generation of tumor escape phenotypes. Cancer Immunol. Immunother..

[7] Del Campo A.B., Kyte J.A., Carretero J., Zinchencko S., Méndez R., González-Aseguinolaza G., Ruiz-Cabello F., Aamdal S., Gaudernack G., Garrido F. (2014). Immune escape of cancer cells with beta2-microglobulin loss over the course of metastatic melanoma. Int. J. Cancer.

[8] Koopman L.A., Corver W.E., Van Der Slik A.R., Giphart M.J., Fleuren G.J. (2000). Multiple genetic alterations cause frequent and heterogeneous human histocompatibility leukocyte antigen class I loss in cervical cancer. J. Exp. Med..

[9] Grasso C.S., Giannakis M., Wells D.K., Hamada T., Mu X.J., Quist M., Nowak J.A., Nishihara R., Qian Z.R., Inamura K. (2018). Genetic mechanisms of immune evasion in colorectal cancer. Cancer Discov..

[10] Aptsiauri N., Carretero R., Garcia-Lora A., Real L.M., Cabrera T., Garrido F. (2008). Regressing and progressing metastatic lesions: Resistance to immunotherapy is predetermined by irreversible HLA class I antigen alterations. Cancer Immunol. Immunother..

[11] Garrido F., Cabrera T., Aptsiauri N. (2010). “Hard” and “soft” lesions underlying the HLA class I alterations in cancer cells: Implications for immunotherapy. Int. J. Cancer.

[12] Zaretsky J.M., Garcia-Diaz A., Shin D.S., Escuin-Ordinas H., Hugo W., Hu-Lieskovan S., Torrejon D.Y., Abril-Rodriguez G., Sandoval S., Barthly L. (2016). Mutations Associated with Acquired Resistance to PD-1 Blockade in Melanoma. N. Engl. J. Med..

[13] Chowell D., Morris L.G.T., Grigg C.M., Weber J.K., Samstein R.M., Makarov V., Kuo F., Kendall S.M., Requena D., Riaz N. (2018). Patient HLA class I genotype influences cancer response to checkpoint blockade immunotherapy. Science.

[14] McGranahan N., Rosenthal R., Hiley C.T., Rowan A.J., Watkins T.B.K., Wilson G.A., Birkbak N.J., Veeriah S., Van Loo P., Herrero J. (2017). Allele-Specific HLA Loss and Immune Escape in Lung Cancer Evolution. Cell.

[15] Garrido F., Perea F., Bernal M., Sánchez-Palencia A., Aptsiauri N., Ruiz-Cabello F. (2017). The Escape of Cancer from T Cell-Mediated Immune Surveillance: HLA Class I Loss and Tumor Tissue Architecture. Vaccines.

[16] Perea F., Sánchez-Palencia A., Gómez-Morales M., Bernal M., Concha Á., García M.M., González-Ramírez A.R., Kerick M., Martin J., Garrido F. (2018). HLA class I loss and PD-L1 expression in lung cancer: Impact on T-cell infiltration and immune escape. Oncotarget.

[17] Perea F., Bernal M., Sánchez-Palencia A., Carretero J., Torres C., Bayarri C., Gómez-Morales M., Garrido F., Ruiz-Cabello F. (2017). The absence of HLA class I expression in non-small cell lung cancer correlates with the tumor tissue structure and the pattern of T cell infiltration. Int. J. Cancer.

[18] Flores-Martín J.F., Perea F., Exposito-Ruiz M., Carretero F.J., Rodriguez T., Villamediana M., Ruiz-Cabello F., Garrido F., Cózar-Olmo J.M., Aptsiauri N. (2019). A Combination of Positive Tumor HLA-I and Negative PD-L1 Expression Provides an Immune Rejection Mechanism in Bladder Cancer. Ann. Surg. Oncol..

[19] Hegde P.S., Karanikas V., Evers S. (2016). The where, the when, and the how of immune monitoring for cancer immunotherapies in the era of checkpoint inhibition. Clin. Cancer Res..

[20] Sweis R.F., Spranger S., Bao R., Paner G.P., Stadler W.M., Steinberg G., Gajewski T.F. (2016). Molecular drivers of the non- T-cell-inflamed tumor microenvironment in urothelial bladder cancer. Cancer Immunol. Res..

[21] Syn N.L., Teng M.W.L., Mok T.S.K., Soo R.A. (2017). De-novo and acquired resistance to immune checkpoint targeting. Lancet Oncol..

[22] Mariathasan S., Turley S.J., Nickles D., Castiglioni A., Yuen K., Wang Y., Kadel E.E., Koeppen H., Astarita J.L., Cubas R. (2018). TGFβ attenuates tumour response to PD-L1 blockade by contributing to exclusion of T cells. Nature.

[23] Van Allen E.M., Miao D., Schilling B., Shukla S.A., Blank C., Zimmer L., Sucker A., Hillen U., Foppen M.H.G., Goldinger S.M. (2015). Genomic correlates of response to CTLA-4 blockade in metastatic melanoma. Science.

[24] Sharma P., Hu-Lieskovan S., Wargo J.A., Ribas A. (2017). Primary, Adaptive, and Acquired Resistance to Cancer Immunotherapy. Cell.

[25] Montes P., Kerick M., Bernal M., Hernández F., Jiménez P., Garrido P., Márquez A., Jurado M., Martin J., Garrido F. (2018). Genomic loss of HLA alleles may affect the clinical outcome in low-risk myelodysplastic syndrome patients. Oncotarget.

[26] Ramal L.M., Maleno I., Cabrera T., Collado A., Ferron A., Lopez-Nevot M.A., Garrido F. (2000). Molecular strategies to define HLA haplotype loss in microdissected tumor cells. Hum. Immunol..

[27] Garrido M.A., Rodriguez T., Zinchenko S., Maleno I., Ruiz-Cabello F., Concha Á., Olea N., Garrido F., Aptsiauri N. (2018). HLA class I alterations in breast carcinoma are associated with a high frequency of the loss of heterozygosity at chromosomes 6 and 15. Immunogenetics.

[28] Castro A., Ozturk K., Pyke R.M., Xian S., Zanetti M., Carter H. (2019). Elevated neoantigen levels in tumors with somatic mutations in the HLA-A, HLA-B, HLA-C and B2M genes. BMC Med. Genom..

[29] Watson N.F.S., Ramage J.M., Madjd Z., Spendlove I., Ellis I.O., Scholefield J.H., Durrant L.G. (2006). Immunosurveillance is active in colorectal cancer as downregulation but not complete loss of MHC class I expression correlates with a poor prognosis. Int. J. Cancer.

[30] Romero J.M., Jiménez P., Cabrera T., Cózar J.M., Pedrinaci S., Tallada M., Garrido F., Ruiz-Cabello F. (2005). Coordinated downregulation of the antigen presentation machinery and HLA class I/β2-microglobulin complex is responsible for HLA-ABC loss in bladder cancer. Int. J. Cancer.

[31] Spranger S., Bao R., Gajewski T.F. (2015). Melanoma-intrinsic β-catenin signalling prevents anti-tumour immunity. Nature.

[32] Kalluri R. (2016). The biology and function of fibroblasts in cancer. Nat. Rev. Cancer.

[33] Peng W., Chen J.Q., Liu C., Malu S., Creasy C., Tetzlaff M.T., Xu C., McKenzie J.A., Zhang C., Liang X. (2016). Loss of PTEN promotes resistance to T cell–mediated immunotherapy. Cancer Discov..

[34] Takahashi H., Sakakura K., Kudo T., Toyoda M., Kaira K., Oyama T., Chikamatsu K. (2017). Cancer-associated fibroblasts promote an immunosuppressive microenvironment through the induction and accumulation of protumoral macrophages. Oncotarget.

[35] García-Rocha R., Moreno-Lafont M., Mora-García M.L., Weiss-Steider B., Montesinos J.J., Piña-Sánchez P., Monroy-García A. (2015). Mesenchymal stromal cells derived from cervical cancer tumors induce TGF-β1 expression and IL-10 expression and secretion in the cervical cancer cells, resulting in protection from cytotoxic T cell activity. Cytokine.

[36] Erdogan B., Webb D.J. (2017). Cancer-associated fibroblasts modulate growth factor signaling and extracellular matrix remodeling to regulate tumor metastasis. Biochem. Soc. Trans..

[37] Carretero R., Cabrera T., Gil H., Saenz-Lopez P., Maleno I., Aptsiauri N., Cozar J.M., Garrido F. (2011). Bacillus Calmette-Guerin immunotherapy of bladder cancer induces selection of human leukocyte antigen class I-deficient tumor cells. Int. J. Cancer.

[38] Jiménez P., Cantón J., Collado A., Cabrera T., Serrano A., Real L.M., García A., Ruiz-Cabello F., Garrido F. (1999). Chromosome loss is the most frequent mechanism contributing to HLA haplotype loss in human tumors. Int. J. Cancer.

[39] López S., Lim E.L., Horswell S., Haase K., Huebner A., Dietzen M., Mourikis T.P., Watkins T.B.K., Rowan A., Dewhurst S.M. (2020). Interplay between whole-genome doubling and the accumulation of deleterious alterations in cancer evolution. Nat. Genet..

[40] Patel S.P., Kurzrock R. (2015). PD-L1 Expression as a Predictive Biomarker in Cancer Immunotherapy. Mol. Cancer Ther..

[41] Wang B., Pan W., Yang M., Yang W., He W., Chen X., Bi J., Jiang N., Huang J., Lin T. (2019). Programmed death ligand-1 is associated with tumor infiltrating lymphocytes and poorer survival in urothelial cell carcinoma of the bladder. Cancer Sci..

[42] Boorjian S.A., Sheinin Y., Crispen P.L., Farmer S.A., Lohse C.M., Kuntz S.M., Leibovich B.C., Kwon E.D., Frank I. (2008). T-Cell Coregulatory Molecule Expression in Urothelial Cell Carcinoma: Clinicopathologic Correlations and Association with Survival. Clin. Cancer Res..

[43] Bellmunt J., Mullane S.A., Werner L., Fay A.P., Callea M., Leow J.J., Taplin M.E., Choueiri T.K., Hodi F.S., Freeman G.J. (2015). Association of PD-L1 expression on tumor-infiltrating mononuclear cells and overall survival in patients with urothelial carcinoma. Ann. Oncol..

[44] Bochner B.H., Hansel D.E., Efstathiou J.A., Konety B., Lee C.T., MacKiernan J.M., Plimack E.R., Reuter V.E., Sridhar S., Vikram R., Amin M.B., Edge S.B., Greene F.B., Byrd D.R., Brook-land R.K., Washington M.K., Gershenwald J.E., Compton C.C., Hess K.R., Sullivan D.C. (2017). Urinary Bladder. AJCC Cancer Staging Manual.

[45] Soukup V., Čapoun O., Cohen D., Hernández V., Babjuk M., Burger M., Compérat E., Gontero P., Lam T., MacLennan S. (2017). Prognostic Performance and Reproducibility of the 1973 and 2004/2016 World Health Organization Grading Classification Systems in Non–muscle-invasive Bladder Cancer: A European Association of Urology Non-muscle Invasive Bladder Cancer Guidelines Panel Syst. Eur. Urol..

[46] Lozano F., Santos-Aguado J., Borche L., Places L., Doménech N., Gayá A., Vilella R., Vives J. (1989). Identification of the amino acid residues defining an intralocus determinant in the α1 domain of HLA-A molecules. Immunogenetics.

[47] Lozano F., Borche L., Places L., Alberola-Ila J., Gayá A., Vilella R., Vives J. (1990). Biochemical and serological characterization of a public antigenic determinant present on HLA-B molecules. Tissue Antigens.

[48] Eckstein M., Erben P., Kriegmair M.C., Worst T.S., Weiß C.-A., Wirtz R.M., Wach S., Stoehr R., Sikic D., Geppert C.I. (2019). Performance of the Food and Drug Administration/EMA-approved programmed cell death ligand-1 assays in urothelial carcinoma with emphasis on therapy stratification for first-line use of atezolizumab and pembrolizumab. Eur. J. Cancer.

[49] Bernal M., Concha A., Sáenz-López P., Rodríguez A.I., Cabrera T., Garrido F., Ruiz-Cabello F. (2011). Leukocyte infiltrate in gastrointestinal adenocarcinomas is strongly associated with tumor microsatellite instability but not with tumor immunogenicity. Cancer Immunol. Immunother..

[50] Carretero F.J., del Campo A.B., Flores-Martín J.F., Mendez R., García-Lopez C., Cozar J.M., Adams V., Ward S., Cabrera T., Ruiz-Cabello F. (2016). Frequent HLA class I alterations in human prostate cancer: Molecular mechanisms and clinical relevance. Cancer Immunol. Immunother..

[51] Garrido F., Cabrera T., Accolla R.S., Bensa J.C., Bodmer W., Dohr G., Drouel M., Fauchel R., Ferrara G.B., Ferrone S., Charron D. (1997). HLA and cancer: 12th International Histocompatibility Workshop study. Genetic Diversity of HLA: Functional and Medical Implication, Proceedings of the Twelfth International Histocompatibility Workshop and Conference.

[52] Montes P., Bernal M., Campo L.N., González-Ramírez A.R., Jiménez P., Garrido P., Jurado M., Garrido F., Ruiz-Cabello F., Hernández F. (2019). Tumor genetic alterations and features of the immune microenvironment drive myelodysplastic syndrome escape and progression. Cancer Immunol. Immunother..

[53] Betensky M., Babushok D., Roth J.J., Mason P.J., Biegel J.A., Busse T.M., Li Y., Lind C., Papazoglou A., Monos D. (2016). Clonal evolution and clinical significance of copy number neutral loss of heterozygosity of chromosome arm 6p in acquired aplastic anemia. Cancer Genet..

[54] Jasek M., Gondek L.P., Bejanyan N., Tiu R., Huh J., Theil K.S., O’Keefe C., McDevitt M.A., Maciejewski J.P. (2010). TP53 mutations in myeloid malignancies are either homozygous or hemizygous due to copy number-neutral loss of heterozygosity or deletion of 17p. Leukemia.

