# Supplementary material for: Tumor Escape Phenotype in Bladder Cancer Is Associated with Loss of HLA Class I Expression, T-Cell Exclusion and Stromal Changes
Source: Int J Mol Sci. 2021 Jul 6;22(14):7248. doi: 10.3390/ijms22147248 (PMC8307653; doi:10.3390/ijms22147248)
Supplement: Supplementary file 1 [file ijms-22-07248-s001.zip › Supplementary Figure S1.pdf]

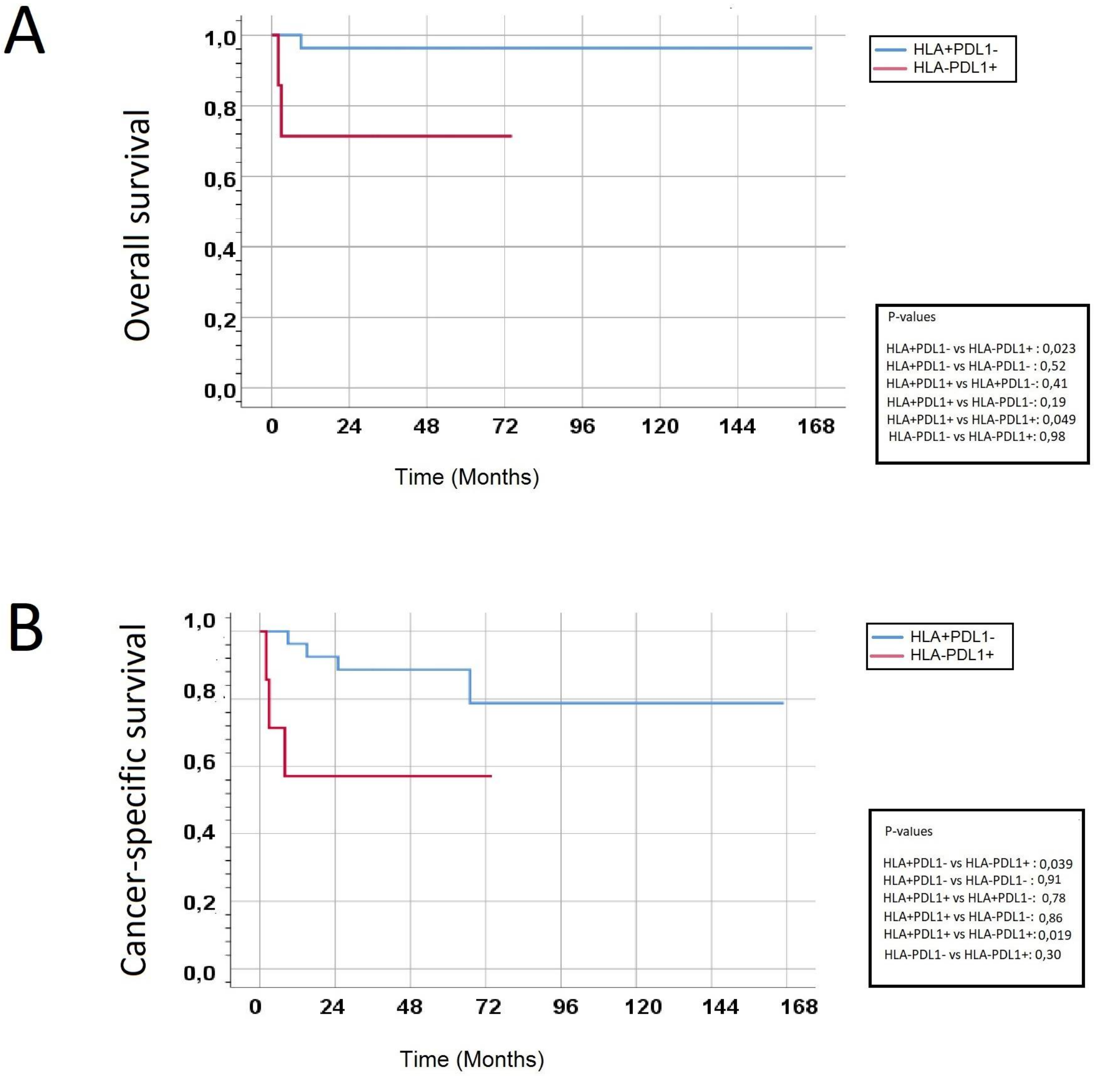

Figure S1. (A) Kaplan-Meier overall and (B) cancer-specific survival curves for patients with bladder cancer, HLA-I+/PD-L1- vs HLA-I-/PD-L1+ phenotypes. P-values were determined using the Log-Rank test. The value $p < 0.05$ was considered statistically significant.
