# Supplementary material for: Tumor Escape Phenotype in Bladder Cancer Is Associated with Loss of HLA Class I Expression, T-Cell Exclusion and Stromal Changes
Source: Int J Mol Sci. 2021 Jul 6;22(14):7248. doi: 10.3390/ijms22147248 (PMC8307653; doi:10.3390/ijms22147248)
Supplement: Supplementary file 1 [file ijms-22-07248-s001.zip › Supplementary Figure S2.pdf]

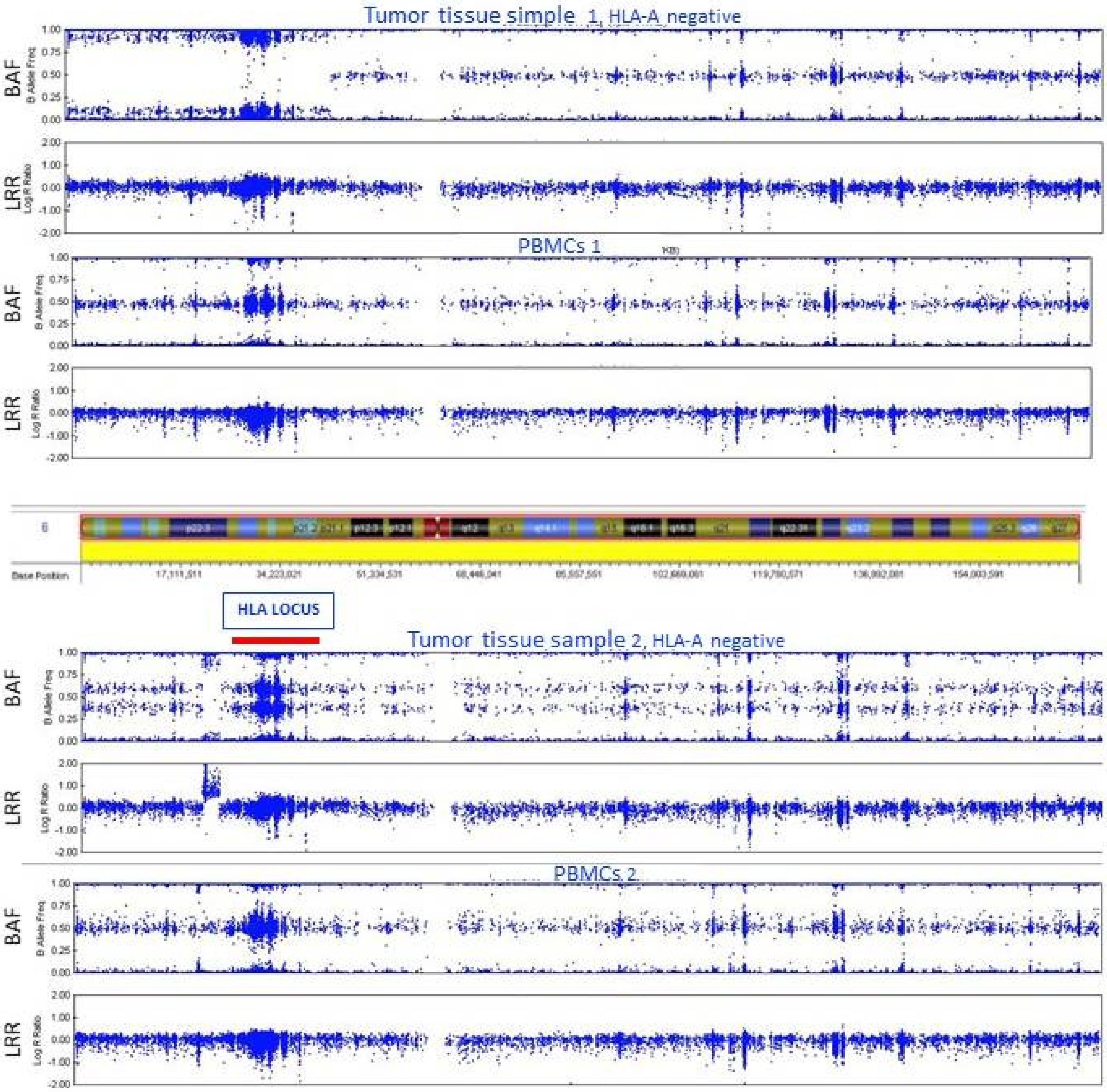

Figure S2. Examples of LOH at chromosome 6 using SNP-array analysis in two tumors with a selective HLA-A locus loss as detected by immunohistochemistry. B-allele frequency (BAF) and long R ratio LRR on the Y-axis indicate three genotypes: BB(BAF=1), AB(BAF=0,5) and AA(BAF=0) and estimated copy number, respectively (LRR plot). The position of HLA genes in the 6p arm of the chromosome 6 is shown. The DNA from Sample 1 showed LOH with a complete loss of all SNPs with AB genotype in the HLA region (area 6pterp21.1) of chromosome 6. Furthermore, loss of SNPs in the AB together with the absence of the copy number alteration (LRR), is indicative of a copy neutral LOH (CN-LOH). The BAF plot obtained for tumor Sample 2 shows the presence of heterogeneity in the analyzed microdissected tissue sample with one clone showing a complete absence of a the entire chromosome 6. Alternatively, these results can be explained by the presence of normal stroma cells in the sample. PBMCs, Peripheral blood mononuclear cells
