# Supplementary material for: Tumor Escape Phenotype in Bladder Cancer Is Associated with Loss of HLA Class I Expression, T-Cell Exclusion and Stromal Changes
Source: Int J Mol Sci. 2021 Jul 6;22(14):7248. doi: 10.3390/ijms22147248 (PMC8307653; doi:10.3390/ijms22147248)
Supplement: Supplementary file 1 [file ijms-22-07248-s001.zip › Supplementary Figure S3.pdf]

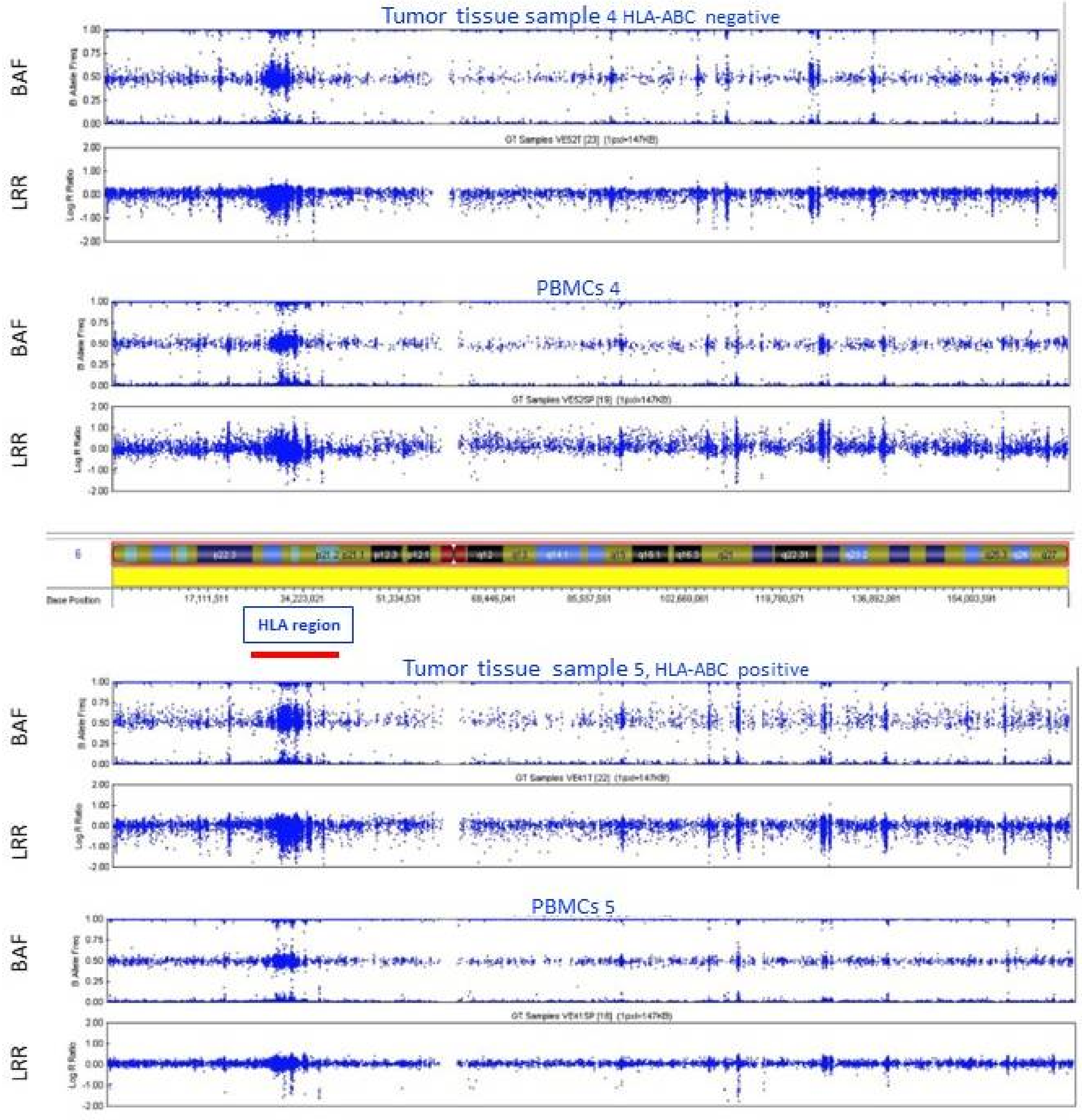

Figure S3. Two examples of SNP-array analysis demonstrating an absence of LOH at chromosome 6 in two samples: one with total HLA class I loss (Sample 4) and another one with normal HLA-I expression (Sample 5).
